# Protective Effect of Mitophagy Regulated by mTOR Signaling Pathway in Liver Fibrosis Associated with Selenium

**DOI:** 10.3390/nu14122410

**Published:** 2022-06-10

**Authors:** Lichun Qiao, Ziwei Guo, Haobiao Liu, Jiaxin Liu, Xue Lin, Huan Deng, Xuan Liu, Yan Zhao, Xiang Xiao, Jian Lei, Jing Han

**Affiliations:** 1 Department of Occupational and Environmental Health, School of Public Health, Health Science Center, Xi’an Jiaotong University, Xi’an 710061, China; 3120115091@stu.xjtu.edu.cn (L.Q.); 3120315064@stu.xjtu.edu.cn (Z.G.); houbiu@stu.xjtu.edu.cn (H.L.); 3120315063@stu.xjtu.edu.cn (J.L.); 1997601@stu.xjtu.edu.cn (X.L.); dh0205@stu.xjtu.edu.cn (H.D.); liuxuan5241@stu.xjtu.edu.cn (X.L.); zy97133@stu.xjtu.edu.cn (Y.Z.); xiaoxiang979@stu.xjtu.edu.cn (X.X.); 20111020034@fudan.edu.cn (J.L.); 2 Global Health Institute, Health Science Center, Xi’an Jiaotong University, Xi’an 712000, China; 3 Key Lab of Public Health Safety of the Ministry of Education, School of Public Health, Fudan University, Shanghai 200433, China; 4 Key Laboratory of Environment and Genes Related to Diseases, School of Public Health, Health Science Center, Xi’an Jiaotong University, Xi’an 710061, China

**Keywords:** liver fibrosis, mitophagy, energy metabolism, selenium, mTOR signaling pathway

## Abstract

Background: As a central organ of energy metabolism, the liver is closely related to selenium for its normal function and disease development. However, the underlying roles of mitochondrial energy metabolism and mitophagy in liver fibrosis associated with selenium remain unclear. Methods: 28 rats were randomly divided into normal, low-selenium, nano-selenium supplement-1, and supplement-2 groups for a 12-week intervention. We observed pathological and ultrastructural changes in the liver and analyzed the effects of selenium deficiency and nano-selenium supplementation on liver metabolic activities and crucial proteins expression of mammalian target of the rapamycin (mTOR) signaling pathway. Results: Selenium deficiency caused liver pathological damage and fibrosis with the occurrence of mitophagy by disrupting normal metabolic activities; meanwhile, the mTOR signaling pathway was up-regulated to enhance mitophagy to clear damaged mitochondria. Furthermore, nano-selenium supplements could reduce the severity of pathological damage and fibrosis in livers and maintain normal energy metabolic activity. With the increased concentrations of nano-selenium supplement, swelling mitochondria and mitophagy gradually decreased, accompanied by the higher expression of mTOR and phosphorylation-modified mTOR proteins and lower expression of unc-51 like autophagy activating kinase 1 (ULK1) and phosphorylation-modified ULK1 proteins. Conclusions: Mitophagy regulated by the mTOR signaling pathway plays a dual protective role on low-selenium inducing liver fibrosis and nano-selenium supplements preventing liver fibrosis. Mitochondrial energy metabolism plays an important role in these processes as well.

## 1. Introduction

Selenium is an essential nutrient element that plays a crucial role in regulating immune function, anti-oxidant damage, endocrine homeostasis, growth, and the development of tissues and organs in humans. As the most important metabolic organ, the liver is sensitive to selenium [1], and selenium deficiency can be preferentially reflected in the liver, kidneys, and serum [2]. Epidemiological studies have found that reducing serum selenium concentration is closely related to liver fibrosis [3], and liver fibrosis was also observed in the portal region of rats with selenium deficiency intervention [4]. In contrast, appropriate selenium supplements have a protective effect on the liver by increasing glutathione peroxidase (GPX) activity and enhancing the anti-oxidant effects to antagonize liver fibrosis [1,4,5]. Liver fibrosis is a post-injury repair response triggered by chronic injury stimuli to the liver, characterized mainly by abnormal connective tissue hyperplasia [6,7]. As an early pathological change in cirrhosis and primary liver cancer [8,9], fibrosis severely affects the normal physiological functions of the liver.

While producing energy, mitochondria generate reactive oxygen species (ROS), which can cause mitochondrial damage and affect the survival state of cells. Cells can selectively clear damaged mitochondria by autophagy, which is known as mitophagy [10], which is closely related to hepatitis, liver fibrosis, and liver cancer [11,12]. Oxidative stress is an important pathogenic factor in liver inflammation and fibrosis [13], and ROS can enhance oxidative stress and inflammation by inhibiting mitophagy, leading to hepatic steatosis, liver fibrosis, and cirrhosis [14,15]. Nevertheless, activating mitophagy can promote the elimination of senescent and damaged mitochondria, thereby ameliorating liver inflammation and fibrosis in cirrhotic rats by maintaining mitochondrial homeostasis [13]. The mammalian target of the rapamycin (mTOR) signaling pathway plays a key role in initiating mitophagy. Normally, mTOR proteins are in a highly expressed, activated state to maintain normal cellular autophagy inhibition by suppressing unc-51 like autophagy activating kinase 1 (ULK1) expression [16]. When upstream signal molecules inhibit mTOR proteins, it relieves the inhibitory effect on downstream ULK1 proteins, thereby inducing mitophagy [16]. ULK1-deficient mouse models exhibited an inhibited state of mitophagy, leading to many damaged mitochondria being unable to be degraded in the cells [17,18], and normal metabolic activities of cells were severely affected.

At present, studies on the mechanism of mitophagy regulated by the mTOR signaling pathway in liver fibrosis are limited. In the present study, we found that the mitochondria energy metabolism exerts an important role in the relationship between selenium and liver fibrosis. Moreover, the effects of mitophagy on low-selenium inducing liver fibrosis and nano-selenium supplements preventing liver fibrosis were also investigated. Hence, our works would contribute to elucidating the mechanism of liver fibrosis induced by low-selenium and providing a theoretical and experimental basis for the nano-selenium supplement as a potential preventive measure against liver fibrosis.

## 2. Materials and Methods

### 2.1. Animal Grouping and Intervention

28 Sprague-Dawley rats purchased from the Animal Experimental Center of Xi’an Jiaotong University were randomly divided into four groups: normal, low-selenium, nano-selenium supplement-1, and nano-selenium supplement-2 groups. All rats were raised for 12 weeks (w) under controlled environmental conditions (12-h light-dark cycle, temperature 22 ± 2 °C, humidity 55 ± 5%). The standard diet and selenium-deficient diet were produced by Trophic Animal Feed High-tech Co. (Nantong, China) according to the AIN-93G formula. The nano-selenium supplement (chondroitin sulfate nano-selenium) was synthesized and characterized by our team, which has been granted a patent for the invention [19]. The normal group was fed with a standard diet (containing 0.20 mg inorganic selenium/kg), the low-selenium group was fed with a selenium-deficient diet (containing 0.02 mg selenium/kg), and the nano-selenium supplement-1 and supplement-2 groups were fed with a selenium-deficient diet plus a lower dose of nano-selenium supplement (containing 0.10 mg selenium/kg) and a higher dose of nano-selenium supplement (containing 0.20 mg selenium/kg), respectively.

### 2.2. Collection of Serum and Liver Samples

Blood samples were collected and refrigerated overnight at 4 °C, followed by centrifugation at 4000 rpm to separate serum, and stored at −20 °C for selenium concentration detection. After sacrificing the rats, liver samples were harvested and then cut into pieces on ice for further experiments. Part of the liver tissues were collected into 1.5 mL sterile non-enzymatic Eppendorf tubes, placed in liquid nitrogen, and stored at −80 °C for selenium concentration detection and metabolomic analysis. The remaining tissues were transferred into 1.5 mL Eppendorf tubes with 2.5% glutaraldehyde fixative and 50 mL 4.0% paraformaldehyde in phosphate-buffered saline (PBS), subsequently stored at 4 °C for hematoxylin and eosin (HE) staining, Masson staining, immunohistochemistry (IHC) staining, and transmission electron microscope (TEM) observation.

### 2.3. Selenium Concentration Detection

The selenium concentration was determined by an atomic fluorescence spectrometer (AFS-9750, Beijing Haiguang Instrument Co., Ltd., Beijing, China). 0.2 g ± 10% liver and 500 μL serum samples from each rat were put into PTFE cups containing 5 mL mixed acid solution (HNO_3_:HClO_4_ = 4:1) for overnight digestion at room temperature. The next day, samples were heated at 160 °C for digestion to dissolve in solution. Five mL 1:1 HCl was then added at the appearance of white smoke, and the reaction was held at 160 °C for 8 min. Subsequently, the digested samples were transferred to volumetric tubes and fixed to 15 ml with double distilled water and then the tubes were left for 30 min. Finally, selenium concentrations were detected following the manufacturer’s measurement instructions.

### 2.4. HE and Masson Stainings and TEM Observation

After fixation in 2.5% glutaraldehyde fixative and 4.0% paraformaldehyde in PBS, liver tissues were embedded in paraffin, and serial sections were cut with a thickness of 6 μm. Paraffin sections were deparaffinized by immersion in xylene and rehydrated with an ethanol solution of gradually decreasing concentrations (100–70%). According to a standardized procedure, sections were stained with HE or Masson dye solutions. Following rinsing with distilled water, sections were dehydrated using a series of increasing concentrations of ethanol solutions (70–100%). The distance between the portal region and central venous region of liver tissue was used to evaluate the integrity of supporting structures and to reflect the viability of the liver. Furthermore, the Ishak score was used to determine the severity of liver fibrosis [20].

The ultrastructure of liver tissue was observed by TEM (H-7650, Hitachi, Japan). After fixation in 2.5% glutaraldehyde solution, sections were washed with 0.2 mol/L PBS and treated with 1.0% osmium tetroxide fixation solution for two hours. Following dehydration with a gradient concentration of ethanol (30–100%), tissue was embedded in epoxy resin. Subsequently, 50–70 nm slices were obtained from each liver sample using an ultramicrotome (LKB-V, Bromma, Sweden). Finally, the slices were observed by TEM after being stained with uranyl acetate and lead citrate solution.

### 2.5. Metabolomics Analysis

#### 2.5.1. Sample Preparation for Metabolomics Analysis

1 mL mixed extract (methanol:acetonitrile:water = 2:2:1, *v*/*v*/*v*, containing L-2-Chlorophenylalanine internal standard (2 μg/mL)) was added to the 50 mg liver sample, followed by grinding at 35 Hz ultrasound frequency for 4 min, sonication in ice water for 5 min (repeated twice), and standing for an hour at −40 °C. After centrifugation at 4 °C and 10,000 rpm for 15 min, 200 μL acetonitrile (50%) was added to 400 μL supernatant with vortex mixing for 30 s and sonication in ice water for 10 min. The clear supernatant was obtained for detection after centrifugation at 4 °C and 13,000 rpm for 15 min. Quality control samples were prepared and analyzed to monitor the stability of instrumental acquisition and evaluate the reliability of experiment data. 

#### 2.5.2. LC-MS/MS Metabolomics Analysis and Data Processing

Ultra-performance liquid chromatography (UPLC, Agilent 1290, Santa Clara, CA, USA) was performed for chromatographic separation of liver metabolites on an Acquity UPLC BEH Amide L.C. column set at 25 °C, with a 0.5 mL/min flow rate and a 2 μL injection volume. Mobile phase A contained 25 mmmol/L ammonium acetate and 25 mmmol/L ammonium hydroxide, and mobile phase B was acetonitrile. Mass spectrometry data were acquired using a TripleTOF 6600 high-resolution mass spectrometer (AB Sciex, Boston, MA, USA) in information-dependent acquisition mode using Analyst^®^ TF 1.7 software (AB Sciex, Boston, MA, USA). ProteoWizard was applied to convert the raw mass spectrometry data into mzXML format, and XCMS software was used for retention time correction, peak identification, peak extraction, peak integration, and peak alignment.

#### 2.5.3. Inter-Group Variation Analysis and Differential Metabolite Screening

The multivariate statistical analysis method of orthogonal partial least-squares discriminant analysis (OPLS-DA) was primarily used to analyze the differences between study groups and obtain the variable importance for the projection (VIP) values. Three parameters (R^2^X, R^2^Y, and Q^2^Y) were used to evaluate the quality of the model. R^2^X and R^2^Y were used to quantify the goodness of fit, while Q^2^Y was used to assess the model’s predictability. The quality of the OPLS-DA model was tested by the permutation test to assess whether the model developed exhibited over-fitting. Fold change (FC) analysis and a Student’s *t*-test were used to analyze the differences in metabolites between the two groups. FC > 1.2, *p*-value < 0.05, and VIP > 1 were jointly considered screening criteria for potential differential metabolites, and volcano plots were used for visual presentation.

#### 2.5.4. Bioinformatics Analysis of Differential Metabolites

Differential metabolites were annotated by the Kyoto Encyclopedia of Genes and Genomes (KEGG) database. The annotated differential metabolites were then classified using the metabolite classification information provided by the Human Metabolome Database (HMDB) database. KEGG pathway enrichment analysis was performed on the annotated differential metabolites, and Fisher’s exact test was used to calculate the significance level of metabolite enrichment for each pathway. The rich factors of each pathway metabolite were also calculated, and the top 20 pathways ranked by the rich factors were presented in bubble plots.

### 2.6. IHC Staining

IHC staining was performed to investigate the expression levels of mTOR, p-mTOR, ULK1, and phosphorylation-modified ULK1 (p-ULK1). The 6 μm thick sections were dewaxed and dehydrated in a gradient of alcohols, and then endogenous peroxidase activity was quenched using 3% (*w*/*v*) hydrogen peroxide at room temperature for 10 min. After washing with PBS, tissue sections were incubated with 10% (*w*/*v*) normal goat serum for 20 min and then with primary antibody (1:100 dilution) overnight at 4 °C. PBS was used to replace the primary antibody as a negative control. After washing, sections were incubated with horseradish peroxidase-conjugated goat anti-rabbit IgG (1:500 dilution) at 37 °C for 20 min and with horseradish enzyme-labeled streptavidin lysate at 37 °C for 30 min. The sections were then visualized with the DAB kit and counterstained with hematoxylin. Representative regions were photographed using an Olympus BX51 fluorescence microscope (Olympus, Tokyo, Japan). At least 6 images were captured at 100× magnification in each section’s portal and central venous regions. Image J software (NIH, Bethesda, MD, USA) was used to quantify the positive staining area.

### 2.7. Statistical Analysis

Statistical analysis was carried out using SPSS 26.0 (SPSS Inc., Chicago, IL, USA), and the results were visualized by GraphPad Prism 9.0.0 (San Diego, CA, USA) and R program 4.1.0 (Auckland, New Zealand). Since the data cannot satisfy both normality and homogeneity of variance, the Kruskal-Wallis test was used to compare differences among groups. *p*-values of less than 0.05 were considered statistically significant.

## 3. Results

### 3.1. Selenium Concentrations of Serum and Liver Samples

**Figure 1:**
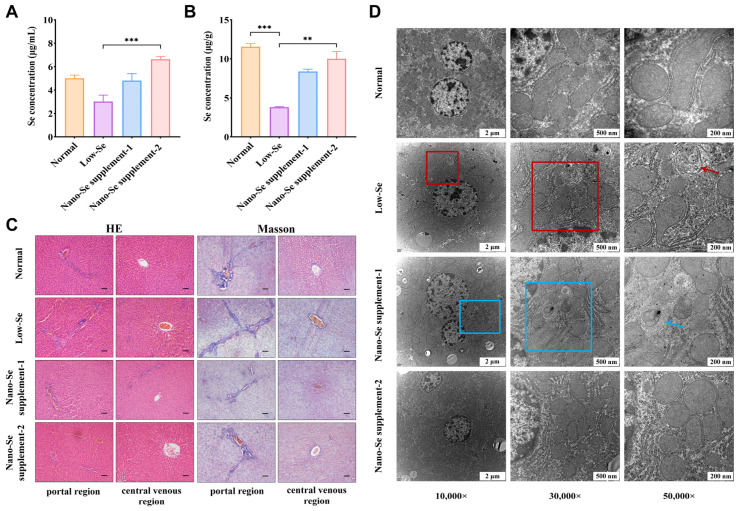
Results of animal experiments after the 12-week intervention. (**A**) Se concentrations of rat serums in different groups. *** *p* < 0.001. (**B**) Se concentrations of rat livers in different groups. ** *p* < 0.01, *** *p* < 0.001. (**C**) Representative HE and Masson staining results in the liver’s portal and central venous regions. Scale bar: 200 μm. (**D**) TEM observations of ultrastructural changes in livers. Compared with the normal group, the malformed mitochondria (**□**) and typical mitophagy phenomenon (**→**) were observed in the low-Se group. There was mild swelling of mitochondria (**□**) and typical mitophagy phenomenon (**→**), but the number was significantly reduced compared with the low-Se group. Se: selenium.

Selenium concentrations were significantly lower in the low-selenium group than in the normal group of serum (*p* = 0.074) and liver (*p* < 0.001) samples. Serum selenium concentrations were significantly higher in the nano-selenium supplement-1 group (*p* = 0.106) and supplement-2 group (*p* < 0.001) than in the low-selenium group. And compared with low-selenium group, liver selenium concentrations were higher in nano-selenium supplement-1 group (*p* = 0.285) and supplement-2 group (*p* < 0.01). In addition, serum selenium concentration was lower in the normal group than in the nano-selenium supplement groups, while liver selenium concentrations were higher in the normal group than in the nano-selenium supplement groups, but the differences were not statistically significant. These results showed that animal models of selenium deficiency and nano-selenium supplementation were successfully constructed (Figure 1A,B).

### 3.2. HE and Masson Staining Results

Pathological changes in the liver of the low-selenium group were presented by HE staining. Compared to the normal group, liver tissue in the low-selenium group was structurally abnormal, showing disorganized hepatic lobules and irregularly arranged hepatocytes. At the same time, abnormal hyperplasia in the portal region and poor continuity of endothelial cells with inflammatory cells clustered in the central venous region were also observed. However, various degrees of improvements in liver tissue structure were observed with different concentrations of the nano-selenium supplement. Hepatic lobules were better structured in the nano-selenium supplement-1 group, and hepatocytes were slightly disorganized but had clear morphology. The endothelial cells were less continuous, and surrounding hepatocytes were slightly swollen in the central venous region. The prevention was more pronounced in the nano-selenium supplement-2 group, without abnormal changes (Figure 1C). The distances between the portal and central venous regions of the liver were shown in Table 1. Compared with the low-selenium group, the distances of normal and nano-selenium supplement groups were longer (*p* < 0.001). The distance of nano-selenium supplement-1 group was shorter than the normal (*p* < 0.001) and nano-selenium supplement-2 (*p* = 0.016) groups. Moreover, the distance of the nano-selenium supplement-2 group was shorter than the normal group, but the difference was not statistically significant.

Masson staining showed more visible fibrosis in the portal region in the low-selenium group than in the normal group, with a tendency to form fibrous bridging to divide the normal hepatic lobules into pathological pseudo lobules. The fibrotic area in the central venous region was also more markedly enlarged. Degrees of liver fibrosis decreased with increased concentrations of nano-selenium supplement intervention. Compared with the low-selenium group, fibrosis in the portal region was reduced in the nano-selenium supplement-1 group, and no prominent fibrosis was observed in the central venous region. No discernible fibrosis was observed in the portal and central venous regions in the nano-selenium supplement-2 group (Figure 1C). Ishak scores to determine the severity of liver fibrosis were shown in Table 1. In the low-selenium group, Ishak scores were statistically higher than the normal group (*p* < 0.001). Compared with the low-selenium group, Ishak scores were lower in the nano-selenium supplement-1 group (*p* = 0.090) and nano-selenium supplement-2 group (*p* < 0.001). These results showed that long-term selenium deficiency could lead to severe liver fibrosis, and nano-selenium supplements could prevent the fibrosis to a certain extent. 

### 3.3. Morphological Changes in the Ultrastructure of Liver

The comparison of ultrastructure changes in the liver among four groups could be observed in Figure 1D. The clarity of the overall structure of hepatocytes in the low-selenium group was less well-defined than in the normal group, with irregular nuclei morphology. Mitochondria appeared atrophied and malformed with typical mitophagy and blurred mitochondrial cristae. In addition, the abnormal aggregation of fibrous connective tissue was observed in the low-selenium group. In contrast, nano-selenium supplement treatments could prevent liver fibrosis to varying degrees, and hepatocytes in both nano-selenium supplement groups had a more defined structure and more regular nuclei than in the low-selenium group. Although abnormal aggregation of connective tissue was also seen in the nano-selenium supplement-1 group, its number and extent were significantly reduced. No abnormal fibrous connective tissue aggregation was observed in the nano-selenium supplement-2 group. Furthermore, slight mitochondrial swelling and typical mitophagy were observed in the nano-selenium supplement-1 group but were significantly reduced compared to the low-selenium group. While in the nano-selenium supplement-2 group, the mitochondria around the nucleus were abundant and clearly structured, and no significant mitochondrial swelling and mitophagy were observed. 

### 3.4. Metabolomics Analysis of Liver Tissue

**Figure 2:**
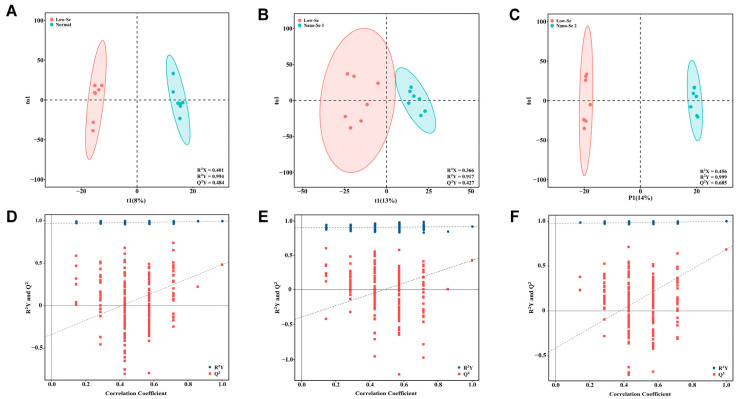
Qualitative analysis of differential metabolites between different groups. (**A**–**C**) OPLS-DA for metabolomics results of liver samples between different groups. (**D**–**F**) Permutation test results of OPLS-DA models between different groups. Se: selenium; OPLS-DA: orthogonal partial least-squares discriminant analysis.

In this study, a total of 2857 metabolites were detected in the liver tissue. OPLS-DA was adopted to observe the metabolic profile changes between groups of rats after different interventions. Parameters of the OPLS-DA model showed a good trend of separation between different intervention groups with clustered distribution characteristics (Figure 2A–C). The permutation test results showed that R^2^ and Q^2^ values on the left were lower than the original R^2^Y and Q^2^ points on the right, indicating that these models were efficient (Figure 2D–F).

**Figure 3:**
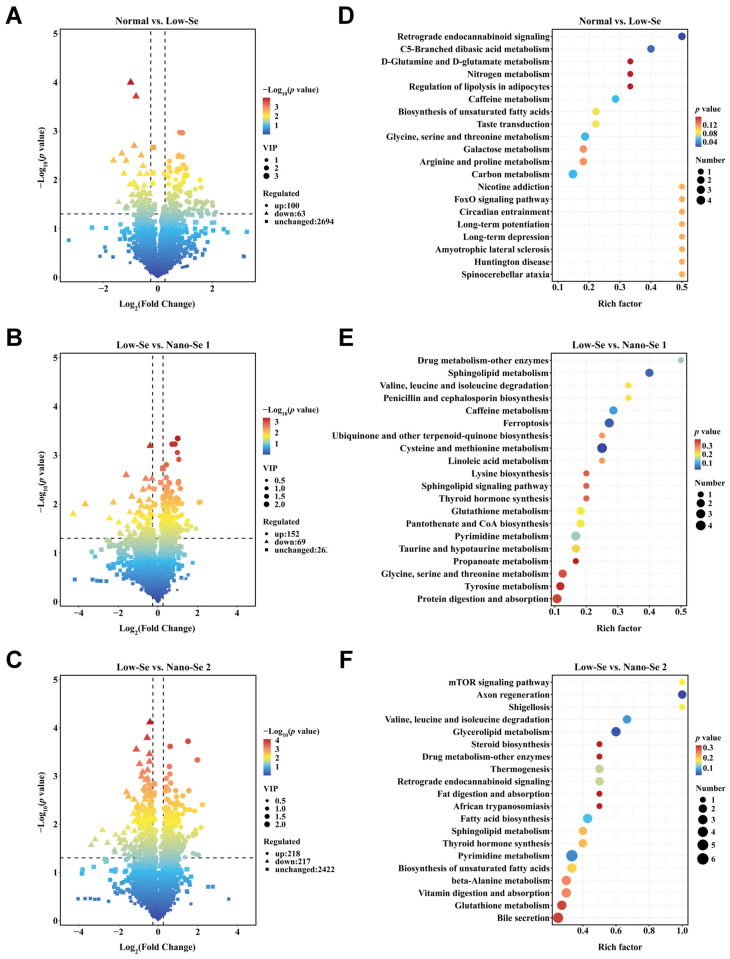
Expression of differential metabolites and KEGG enrichment analysis between different groups. (**A**–**C**) Volcano plots of differential metabolites expression in the normal group vs. low-Se group, low-Se group vs. nano-Se supplement-1 group, and low-Se group vs. nano-Se supplement-2 group. (**D**–**F**) Top 20 rich factors of KEGG enrichment analysis results for metabolites differentially expressed in the normal group vs. low-Se group, low-Se group vs. nano-Se supplement-1 group, and low-Se group vs. nano-Se supplement-2 group. Se: selenium; KEGG: Kyoto Encyclopedia of Genes and Genomes, VIP: variable importance for the projection.

Volcano plots showed the expression of potential differential metabolites screened by screening criteria in different intervention groups. There were 163, 221, and 435 differential metabolites screened between normal and low-selenium groups, low-selenium and nano-selenium supplement-1 groups, and low-selenium and nano-selenium supplement-2 groups, respectively (Figure 3A–C). After KEGG annotation, a total of 16 differential metabolites were identified between normal and low-selenium groups, of which eight were up-regulated and eight were down-regulated. The differential metabolites were mainly classified as fatty acyl, carboxylic acids and derivatives, organic oxygen compounds, and organic nitrogen compounds (Table S1). The enrichment analysis of KEGG revealed that differential metabolites are mainly involved in C5-branched-chain dibasic acid metabolism, D-glutamine and D-glutamate metabolism, regulation of lipolysis in adipocytes, biosynthesis of unsaturated fatty acids, glycine, serine and threonine metabolism, arginine and proline metabolism, galactose metabolism, and carbon metabolism (Figure 3D). These indicated that selenium deficiency could affect liver glucolipid metabolic pathways and amino acid metabolic pathways, which are closely linked to energy metabolism.

Between low-selenium and nano-selenium supplement-1 groups, a total of 20 differential metabolites were identified, of which 13 were up-regulated and seven were down-regulated. The differential metabolites were mainly classified as carboxylic acids and derivatives, organonitrogen compounds, organooxygen compounds, benzene and substituted derivatives, and fatty acyls (Table S2). The enrichment analysis revealed that differential metabolites were mainly associated with sphingolipid metabolism, valine, leucine and isoleucine degradation, cysteine and methionine metabolism, linoleic acid metabolism, lysine biosynthesis, glutathione metabolism, glycine, serine and threonine metabolism, tyrosine metabolism, and protein digestion and absorption (Figure 3E). It was shown that the nano-selenium supplement primarily affects amino acid metabolism and fatty acid metabolic pathways, which were closely linked to energy metabolism, and lower concentrations of nano-Se could affect various energy metabolic processes. 

Furthermore, a total of 49 differential metabolites were identified between low-selenium and nano-selenium supplement-2 groups, of which 18 were up-regulated and 31 were down-regulated. The differential metabolites were mainly classified as carboxylic acids and derivatives, fatty acyls, organonitrogen compounds, and organooxygen compounds (Table S3). The enrichment analysis showed that differential metabolites were mainly related to the valine, leucine and isoleucine degradation, glycerolipid metabolism, steroid biosynthesis, fat digestion and absorption, fatty acid biosynthesis, sphingolipid metabolism, and biosynthesis of unsaturated fatty acids, which were closely linked to energy metabolism (Figure 3F). In addition, the glutathione metabolic signaling pathway was also altered. Remarkably, the mTOR signaling pathway was changed after nano-selenium supplement intervention, and it was highly correlated with energy metabolism and mitophagy.

### 3.5. IHC Staining of mTOR, p-mTOR, ULK1, and p-ULK1

**Figure 4:**
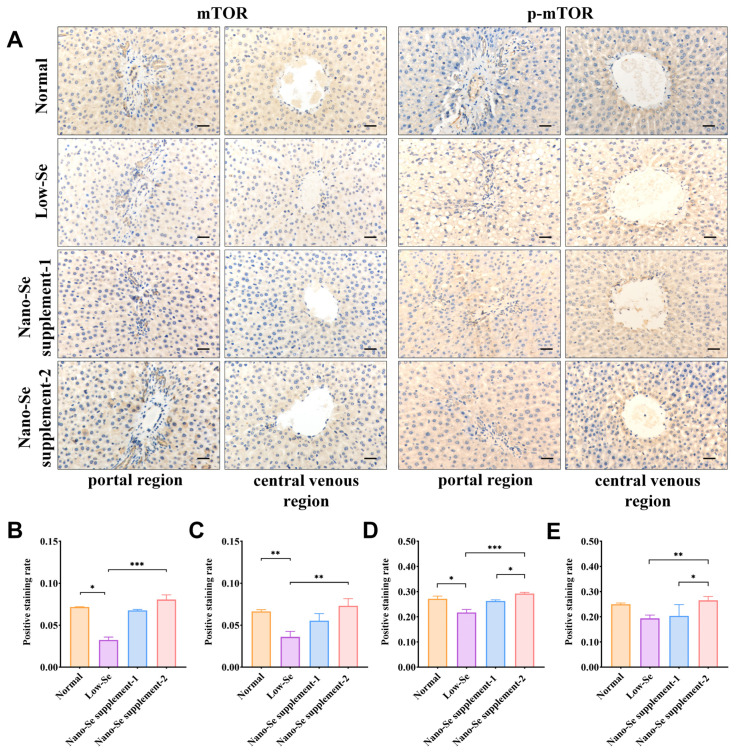
IHC staining results of mTOR and p-mTOR of livers in different groups. (**A**) Representative IHC staining of mTOR and p-mTOR in the portal and central venous regions. (**B**,**C**) The positive staining rates of mTOR in the portal and central venous regions. (**D**,**E**) The positive staining rates of p-mTOR in the portal and central venous regions. Scale bar: 200 μm. * *p* < 0.05, ** *p* < 0.01, *** *p* < 0.001. Se: selenium; mTOR: mammalian target of the rapamycin; p-mTOR: phosphorylation-modified mTOR.

**Figure 5:**
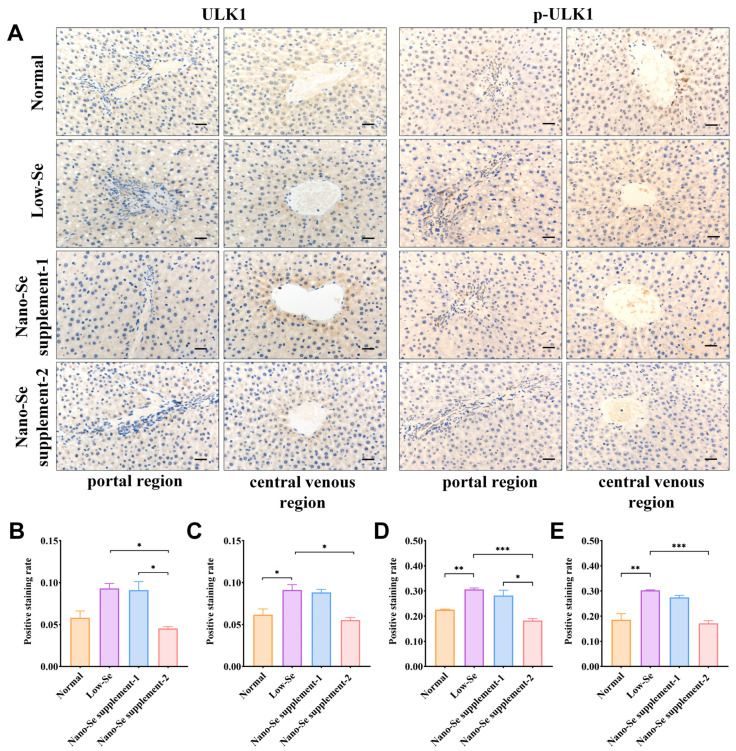
IHC staining results of ULK1 and p-ULK1 of livers in different groups. (**A**) Representative IHC staining of ULK1 and p-ULK1 in the portal and central venous regions. (**B**,**C**) The positive staining rates of ULK1 in the portal and central venous regions. (**D**,**E**) The positive staining rates of p-ULK1 in the portal and central venous regions. Scale bar: 200 μm. * *p* < 0.05, ** *p* < 0.01, *** *p* < 0.001. Se: selenium; ULK1: unc-51 like autophagy activating kinase 1; p-ULK1: Phosphorylation-modified ULK1.

Representative results of IHC staining of mTOR, ULK1, p-mTOR, and p-ULK1 were shown in Figure 4A and Figure 5A. The overall staining was lighter, and the positive staining rates were lower in the portal and central venous regions of low-selenium groups than in normal groups, suggesting that mTOR and p-mTOR proteins were lowly expressed in low-selenium groups. Compared with low-selenium groups, the cytoplasm of the portal and central venous regions in nano-selenium supplement-1 and supplement-2 groups stained darker, and their positive staining rates increased to varying degrees. In addition, the positive staining rates of p-mTOR were statistically higher in the nano-selenium supplement-2 group than in the nano-selenium supplement-1 groups (Figure 4B–E).

In contrast, ULK1 and p-ULK1 showed opposite changes from the mTOR and p-mTOR. Compared with normal groups, the overall staining was more profound, and the positive staining rates were higher in the portal and central venous regions in low-selenium groups, suggesting that ULK1 and p-ULK1 proteins were highly expressed. Moreover, the positive staining rates of ULK1 and p-ULK1 in the nano-selenium supplement-1 groups were close to that of low-selenium groups but higher than that of normal and nano-selenium supplement-2 groups. With increased concentrations of the nano-selenium supplement, the staining depth in the portal and central venous regions gradually decreased, and their positive staining rates also declined (Figure 5B–E).

## 4. Discussion

As the central organ of energy metabolism of carbohydrate, lipid, and amino acids, the liver’s normal function and disease development are closely related to selenium. However, studies on the role of impaired mitochondrial energy metabolism and mitophagy activity in the process of liver fibrosis due to selenium deficiency are limited. The present study provided evidence that mitophagy regulated by the mTOR signaling pathway plays a dual protective role on low-selenium inducing liver fibrosis and nano-selenium supplements preventing liver fibrosis.

As an important organ for regulating selenium concentration in the organism, the liver exhibits a special sensitivity to selenium [1] and synthesizes selenoproteins to maintain normal metabolic activities in the case of selenium deficiency [21]. However, when selenium deficiency exceeds the regulatory capacity of the liver, damage to the liver tissue structure will occur. Similarly, in the present study, selenium deficiency also led to structure disruption of liver tissue and fibrosis. Studies have shown that low-selenium intervention decreases liver GPX4 and selenoprotein P levels and up-regulates inflammatory factors and oxidative stress levels [22,23]. Furthermore, in animal experiments, selenium deficiency can lead to apoptosis of hepatocytes and up-regulate endoplasmic reticulum stress levels [24], leading to detrimental effects on liver histology. Liver fibrosis is mainly accompanied by the disorganization of liver tissues and damage to supporting structures, and the distance between the portal and central venous regions in the low-selenium group could reflect the degree of damage. We also found that moderate nano-selenium supplementation can prevent structural disorders of the liver and has a certain protective effect on liver tissue morphology. The degree of lesions gradually decreased with the increased concentration of nano-selenium supplement. Selenium supplementation alters fatty acid and energy metabolism in the liver [25] and interacts with other elements to reduce the accumulation of toxic heavy metals [25,26], thereby improving liver pathology. Animal experiments suggest that selenium supplementation changes the metabolic profile of the liver and enhances the metabolism of selenoprotein P, tumor suppressor protein P53, thyroid hormone, and peroxisome proliferator-activated receptor γ [27]. Increased anti-oxidant levels may protect against histopathological alterations in the liver and improve liver function.

The most severe liver fibrosis could be seen in the low-selenium group, and the degree of liver fibrosis was progressively reduced with increased concentrations of the nano-selenium supplement. In the study of liver fibrosis models, selenium levels were coherently down-regulated with expression levels of anti-oxidant factors such as selenoprotein P and GPX [28]. Investigation of populations with different severity of cirrhosis revealed the presence of Se deficiency in liver fibrosis [29], which may be associated with the impaired metabolism of selenomethionine [30]. At the same time, up-regulation expression of pro-inflammatory response factors and the pro-fibrotic cytokines Interleukin-6 (IL-6) and growth/differentiation factor-15 (GDF-15) was also observed [31]. The liver of rats with long-term selenium deficiency showed an imbalance in matrix metalloproteinase expression and fibrosis in the venous region [4]. Furthermore, selenium was found to prevent acute alcoholism-induced cirrhosis by up-regulating the expression level of selenoproteins [32]. Selenium-glutathione probiotic can effectively attenuate liver fibrosis by activating SIRT1 signaling and attenuating hepatic oxidative stress, endoplasmic reticulum stress, inflammation, and mitogen-activated protein kinase (MAPK) signaling [33].

The differential metabolites identified between normal and low-selenium groups primarily involved carbohydrates, amino acids, and fatty acids, and the main signaling pathways involved were those related to lipid and amino acid metabolism. Experiments in mice found that selenium deficiency can affect amino acid metabolism [34], and reductions in free fatty acids, mono- and diacylglycerols, and endocannabinoids reflect lipid metabolism disturbance [35]. These are consistent with the present study’s findings, suggesting that long-term selenium deficiency may affect the energy metabolic processes of carbohydrate, lipid, and amino acids in the liver. The KEGG analysis of low-selenium and nano-selenium supplement-1 groups showed that the lower concentration of nano-selenium supplement mainly affects the signaling pathways of liver amino acids and fatty acid metabolism, which are closely related to energy metabolism. In mice, selenium supplementation increases fatty acids (triglycerides, phosphorylcholine, sterols, etc.) and energy metabolism [25]. Moreover, selenium does not act directly through selenoproteins but is closely related to metabolic processes such as glucose transport and fatty acid β-oxidation [36], suggesting that fatty acid metabolism may play an essential role in selenium intervention. The differential metabolites between low-selenium and nano-selenium supplement-2 groups were mainly involved in signaling pathways related to energy metabolism and glutathione metabolic pathways. Population intervention studies found that selenium and coenzyme Q10 interventions alter metabolic pathways of pentose phosphate, mevalonate, β-oxidation, and xanthine oxidase and reduce oxidative stress and inflammatory factors [37]. Additionally, selenium can suppress the increase of lipid peroxidation (LPO), protein carbonyl (PCO), glutathione oxidation, malondialdehyde (MDA), and ROS, prevent the oxidative stress-induced increase in GPX and glutathione reductase (GR) activity and also enhance anti-oxidant capacity, such as an increase in GSH [35,38,39,40], which agrees with the present study. Interestingly, we found that differential metabolites between low-selenium and nano-selenium supplement-2 groups were closely linked to mTOR signaling pathway regulating mitophagy.

Typical mitophagy with many mitochondrial malformations, impaired mitochondrial structure, and blurred mitochondrial cristae morphology was observed in hepatocytes of the low-selenium group compared to the normal group, in keeping with the previous study [41]. Notably, in addition to the liver, selenium deficiency is also closely associated with autophagy in spleen and heart tissues [42,43]. Based on the present study, it can be inferred that long-term low-selenium intervention may induce mitophagy. We also found that nano-selenium supplements could reduce mitochondrial damage, positively correlated with the concentrations. Moreover, higher concentrations of nano-selenium supplement could inhibit mitophagy and significantly prevent mitochondrial structure damage. Selenium supplementation can enhance the anti-oxidant system by inhibiting the ROS and malonaldehyde MDA production, thereby antagonizing the liver and pancreas’s autophagy caused by heavy metal cadmium in animal studies [42,43]. Furthermore, selenium-enriched yeast intervention in mice experiments confirmed that selenium inhibited autophagic activity [44].

The regulation of autophagy is an extremely complex process involving many different signaling pathways. As a significant negative regulator, mTOR performs a pivotal role in autophagy initiation. In this study, low-selenium down-regulated the expression of mTOR and p-mTOR proteins, whereas it up-regulated the expression of ULK1 and p-ULK1 proteins and mitophagy levels. These results suggested that enhanced mitophagy may play a role in repairing damage in liver fibrosis. ULK1 proteins are located downstream of mTOR proteins, and when mTOR proteins are in a state of inhibition, they deregulate the downstream ULK1 proteins, thereby inducing mitophagy [16]. The activation of ULK1 proteins induces activation of hepatic mitophagy activity [45], which in turn attenuated isoproterenol-induced myocardial fibrosis in mice [46]. On the contrary, the expression of mTOR and p-mTOR proteins gradually increased, and mitophagy was reduced in nano-selenium supplement groups, consistent with the other studies [27,45,47], suggesting that selenium can increase mTOR and p-mTOR protein expression and effectively reduce oxidative stress as well as autophagy [41]. Furthermore, the expression of ULK1 and p-ULK1 proteins decreased after nano-selenium supplement interventions. A previous study confirmed that partial inhibition of ULK1 expression impaired the ameliorative effects of resveratrol on hepatic histology, fibrosis, oxidative status, inflammation, and nuclear factor kappa B (NF-κB) activity [48]. These results indicated that when nano-selenium supplements reduce liver fibrosis severity, the mTOR signaling pathway would be down-regulated to suppress mitophagy to avoid abnormally activated mitophagy activity from damaging hepatocytes.

This study investigated the different effects of selenium deficiency and supplementation, allowing a more comprehensive study of the relationship between selenium and liver fibrosis. Meanwhile, the previous experimental studies on selenium mostly focused on oxidative stress and the imbalance of glutathione metabolism. In contrast, the present study focused on energy metabolism disorder and mitophagy, which was somewhat innovative. However, there are also some limitations to this study. Firstly, the key regulatory proteins involved in the mTOR signaling pathway were few, and subsequent studies can focus on more proteins to comprehensively investigate the role of the mTOR signaling pathway in regulating mitophagy in the association between selenium and liver fibrosis. In addition, although this study applied IHC to study the expression of mTOR and Ulk1 proteins, it is still necessary to apply western blots and gene sequencing to verify protein and gene levels. Finally, more concentration gradients should be designed to explore the dose-effect relationship of selenium supplementation comprehensively.

## 5. Conclusions

In conclusion, this study demonstrated that mitophagy regulated by the mTOR signaling pathway plays a dual protective role in liver fibrosis associated with selenium. When severe low-selenium leads to severe damage to liver tissue, the mTOR signaling pathway will be up-regulated to enhance mitophagy, scavenge damaged mitochondria, and maintain the stability of the cellular energy supply system. Conversely, nano-selenium supplements could reduce or prevent the development of liver fibrosis, and the possible mechanism is that the mTOR signaling pathway regulates the down-regulation of mitophagy to avoid the damaging effects of abnormally activated mitophagy on the liver. 

## Figures and Tables

**Table 1:** Distance between the portal and central venous regions of the liver and Ishak score to determine liver fibrosis severity.

Group	Distance (μm)	Ishak Score
Normal	235.68 (208.98, 272.95) ***^, ###^	0 (0, 0) ***
Low-selenium	158.64 (130.11, 182.84)	2 (1, 4)
Nano-selenium supplement-1	187.27 (167.16, 213.64) ***	1 (0, 2.75)
Nano-selenium supplement-2	224.09 (190.45, 266.36) ***^, #^	1 (0, 1) ***

Compared with the low-selenium group: *** *p* < 0.001; compared with the nano-selenium supplement-1 group: ^#^ *p* < 0.05, ^###^ *p* < 0.001.

## Data Availability

Not applicable.

## Supplementary Materials

The following supporting information can be downloaded at: https://www.mdpi.com/article/10.3390/nu14122410/s1, Table S1: Differential metabolites and their HMDB_taxonomy in liver samples from normal and low-selenium groups. Table S2: Differential metabolites and their HMDB_taxonomy in liver samples from low-selenium and nano-selenium supplement-1 groups. Table S3: Differential metabolites and their HMDB_taxonomy in liver samples from low-selenium and nano-selenium supplement-2 groups.
