# Supplementary material for: Protective Effect of Mitophagy Regulated by mTOR Signaling Pathway in Liver Fibrosis Associated with Selenium
Source: Nutrients. 2022 Jun 10;14(12):2410. doi: 10.3390/nu14122410 (PMC9227084; doi:10.3390/nu14122410)
Supplement: Supplementary file 1 [file nutrients-14-02410-s001.zip › nutrients-1766403-supplementary.pdf]

Supplementary Materials

Table S1. Differential metabolites and their HMDB_taxonomy in liver samples from normal and low-selenium groups

No.	Metabolites	FC	*P*_value	VIP	Regulated	HMDB_taxonomy
1	Glycerol	1.33	0.01	2.63	up	Organooxygen compounds
2	O-Acetyl-L-serine	4.00	0.03	2.24	up	Carboxylic acids and derivatives
3	Xanthine	3.49	0.03	2.16	up	Imidazopyrimidines
4	L-homocysteic acid	1.42	0.03	2.11	up	Carboxylic acids and derivatives
5	cis-9-Palmitoleic acid	1.90	0.05	1.98	up	Fatty Acyls
6	Eicosapentaenoic Acid	2.00	0.01	2.55	up	Fatty Acyls
7	Dihomo-gamma-Linolenic Acid	1.44	0.03	2.09	up	Fatty Acyls
8	Ajmalicine	1.38	0.03	1.96	up	--
9	Glyoxylate	0.67	0.04	1.78	down	Carboxylic acids and derivatives
10	Dimethylglycine	0.71	0.03	1.99	down	Carboxylic acids and derivatives
11	L-Glutamate	0.74	0.02	2.11	down	Carboxylic acids and derivatives
12	4-Hydroxyphenylpyruvate	0.65	0.04	2.02	down	Benzene and substituted derivatives
13	Atrolactic acid	0.72	0.05	1.74	down	Phenols
14	3-Phosphoserine	0.49	0.01	2.44	down	Carboxylic acids and derivatives
15	D-Fructose	0.53	0.02	2.34	down	--
16	Phytosphingosine	0.70	0.02	2.09	down	Organonitrogen compounds

HMDB: Human Metabolome Database, FC: Fold change, VIP: variable importance for the projection.

Table S2. Differential metabolites and their HMDB_taxonomy in liver samples from low-selenium and nano-selenium supplement-1 groups

No.	Metabolites	FC	*P*_value	VIP	Regulated	HMDB_taxonomy
1	Phenol	1.29	0.03	1.74	up	--
2	L-Cysteine	1.33	0.02	1.78	up	Carboxylic acids and derivatives
3	Barbituric acid	1.80	0.03	1.55	up	Diazines
4	Xylitol	2.06	0.03	1.47	up	Organooxygen compounds
5	1-Methylxanthine	1.41	0.03	1.64	up	Imidazopyrimidines
6	L-Cysteic acid	1.20	0.04	1.62	up	Carboxylic acids and derivatives
7	4-Hydroxyphenylpyruvate	1.98	0.00	1.90	up	Benzene and substituted derivatives
8	N-Acetylaspartate	1.74	0.02	2.01	up	Carboxylic acids and derivatives
9	Methylmalonic acid	2.72	0.03	1.79	up	Carboxylic acids and derivatives
10	L-Cystine	1.28	0.04	1.62	up	Carboxylic acids and derivatives
11	L-Saccharopine	1.59	0.05	1.56	up	Carboxylic acids and derivatives
12	Phytosphingosine	1.65	0.00	2.28	up	Organonitrogen compounds
13	Sphinganine	1.40	0.02	1.75	up	Organonitrogen compounds
14	Xanthine	0.25	0.02	1.89	down	Imidazopyrimidines
15	Dihydrouracil	0.59	0.04	1.73	down	Diazines
16	1,2-Diacetylhydrazine	0.59	0.04	1.92	down	Organonitrogen compounds
17	Porphobilinogen	0.45	0.04	1.70	down	Organonitrogen compounds
18	Creatine	0.54	0.03	1.49	down	Carboxylic acids and derivatives
19	Dihomo-gamma-Linolenic Acid	0.60	0.01	2.25	down	Fatty Acyls
20	Glutathione	0.37	0.04	1.81	down	Carboxylic acids and derivatives

HMDB: Human Metabolome Database, FC: Fold change, VIP: variable importance for the projection.

Table S3. Differential metabolites and their HMDB_taxonomy in liver samples from low-selenium and nano-selenium supplement-2 groups

No.	Metabolites	FC	*P*_value	VIP	Regulated	HMDB_taxonomy
1	Dihydroxyacetone	1.71	0.03	1.62	up	Organooxygen compounds
2	Dimethylglycine	1.32	0.01	1.69	up	Carboxylic acids and derivatives
3	Glyceric acid	1.50	0.03	1.51	up	Organooxygen compounds
4	Cytosine	1.21	0.02	1.74	up	Diazines
5	Taurine	1.37	0.03	1.57	up	Organic sulfonic acids and derivatives
6	Theobromine	1.38	0.03	1.63	up	Imidazopyrimidines
7	Phosphorylcholine	1.30	0.01	1.87	up	Organonitrogen compounds
8	Methylmalonic acid	3.38	0.04	1.54	up	Carboxylic acids and derivatives
9	D-Fructose	1.56	0.03	1.67	up	--
10	L-Saccharopine	2.03	0.00	1.99	up	Carboxylic acids and derivatives
11	Pyridoxal 5'-phosphate	1.66	0.04	1.46	up	Pyridines and derivatives
12	Phytosphingosine	1.51	0.01	1.77	up	Organonitrogen compounds
13	Sphinganine	1.48	0.01	1.90	up	Organonitrogen compounds
14	Alpha-N-Phenylacetyl-L-glutamine	1.40	0.05	1.40	up	Carboxylic acids and derivatives
15	Erucic acid	1.60	0.04	1.43	up	Fatty Acyls
16	Arachidonoyl Ethanolamide	1.41	0.02	1.65	up	Organonitrogen compounds
17	Estrone 3-sulfate	1.40	0.02	1.60	up	Steroids and steroid derivatives
18	Folate	1.35	0.01	1.91	up	Carboxylic acids and derivatives
19	Glycerol	0.82	0.03	1.58	down	Organooxygen compounds
20	Pyrrole-2-carboxylic acid	0.52	0.00	1.98	down	Pyrroles
21	Indole	0.77	0.00	1.92	down	Indoles and derivatives

22	Barbituric acid	0.81	0.02	1.73	down	Diazines
23	L-Leucine	0.78	0.01	1.86	down	Carboxylic acids and derivatives
24	Tyramine	0.10	0.03	1.71	down	Benzene and substituted derivatives
25	Xanthine	0.26	0.02	1.73	down	Imidazopyrimidines
26	p-Hydroxyphenylacetic acid	0.53	0.01	1.78	down	Phenols
27	Dihydrouracil	0.52	0.02	1.81	down	Diazines
28	1,2-Diacetylhydrazine	0.58	0.03	1.62	down	Organonitrogen compounds
29	N6-Acetyl-L-lysine	0.80	0.03	1.54	down	Carboxylic acids and derivatives
30	2'-Deoxy-D-ribose	0.57	0.01	1.90	down	Organooxygen compounds
31	Dodecanoic acid	0.64	0.00	1.97	down	Steroids and steroid derivatives
32	L-Tryptophan	0.75	0.00	1.91	down	Indoles and derivatives
33	N-Acetyl-L-phenylalanine	0.77	0.01	1.72	down	Carboxylic acids and derivatives
34	5-L-Glutamyl-L-alanine	0.63	0.03	1.59	down	Carboxylic acids and derivatives
35	L-Carnosine	0.55	0.03	1.71	down	Peptidomimetics
36	2'-Deoxyuridine	0.43	0.00	2.07	down	Pyrimidine nucleosides
37	Myristic acid	0.75	0.01	1.78	down	Fatty Acyls
38	Porphobilinogen	0.39	0.03	1.71	down	Organonitrogen compounds
39	cis-9-Palmitoleic acid	0.46	0.02	1.74	down	Fatty Acyls
40	3-Ureidopropionate	0.76	0.01	1.74	down	Organic carbonic acids and derivatives
41	16-Hydroxypalmitic acid	0.68	0.00	2.31	down	Fatty Acyls
42	2-Deoxyribose 5-phosphate	0.75	0.02	1.57	down	Organooxygen compounds
43	Eicosapentaenoic Acid	0.52	0.01	1.88	down	Fatty Acyls
44	Dihomo-gamma-Linolenic Acid	0.68	0.02	1.71	down	Fatty Acyls
45	Glutathione	0.31	0.03	1.67	down	Carboxylic acids and derivatives

46	Ajmalicine	0.67	0.01	1.80	down	--
47	20-hydroxy LTB4	0.48	0.05	1.54	down	Fatty Acyls
48	Glutathione disulfide	0.66	0.01	1.92	down	Carboxylic acids and derivatives

HMDB: Human Metabolome Database, FC: Fold change, VIP: variable importance for the projection.
